# Self-related competence and goal orientation form the strongest cross-construct connection between social and emotional competence and academic resilience in university students: a dimension-level network analysis

**DOI:** 10.3389/fpsyg.2026.1966171

**Published:** 2026-10-02

**Authors:** Runiu Zhu, Bohan Wang, Xiaofei Chen

**Affiliations:** School of Educational Science, Anqing Normal University, Anqing, China

**Keywords:** academic resilience, bridge strength, goal orientation, network analysis, self-regulation, social and emotional competence, university students

## Abstract

**Introduction:**

This study aimed to examine conditional associations between social and emotional competence and academic resilience among university students and to identify dimensions with the strongest overall and cross-construct connectivity.

**Methods:**

In a cross-sectional survey, 1,191 students from six universities in Anhui Province, China, completed self-report questionnaires. An EBICglasso network was estimated from Spearman correlations among four social and emotional competence dimensions and six academic resilience dimensions. Node strength, bridge strength, and bootstrap accuracy and stability were assessed.

**Results:**

The network retained 28 of 45 possible edges, including nine cross-construct connections. The strongest cross-construct edges linked self-related competence with goal orientation (0.37), other-related competence with acceptance (0.29), and collective-related competence with meaning orientation (0.24). Goal orientation had the highest strength (0.95), while self-related competence had the highest bridge strength among the social and emotional competence dimensions (0.44). Both centrality indices had correlation-stability coefficients of 0.75. The three leading cross-construct edges remained positive and retained their rankings across alternative tuning parameters and the unregularized partial-correlation network.

**Discussion:**

In this sample, self-regulatory and relational competence dimensions showed distinct patterns of association with academic resilience facets. These findings support examining specific dimension pairs and provide hypotheses for longitudinal research on academic adaptation.

## 1 Introduction

University study places students in learning environments characterized by greater autonomy, changing routines, fluctuating workloads, performance pressure, and uncertainty about future educational and career pathways. Setbacks in this context can interrupt engagement and make sustained effort difficult, particularly when students must organize their learning with less external structure. Academic resilience refers to the capacity to sustain or recover adaptive academic functioning when facing difficulty, failure, or prolonged pressure (Cassidy, 2016; Martin and Marsh, 2006, 2008). It draws on motivational, cognitive, emotional, and self-regulatory processes that support continued participation in learning. Recent university research has located academic resilience within a broader resource system: study-related resources and self-efficacy have been associated with resilience, engagement, and well-being, while prospective evidence from Chinese university students has linked earlier self-efficacy, motivation, and social support with later academic resilience (Bagdžiūnienė et al., 2025; Yu et al., 2026). These findings make resilience a central issue in understanding adaptation to the demands of higher education. They also show that resilience is not simply a stable personal attribute: it is related to the resources available within students and their study environments.

Social and emotional competence (SEC) offers one perspective on the resources students bring to these demands. SEC concerns how individuals understand and manage relationships with themselves, other people, and collective groups while making responsible decisions. Among Chinese university students, Chen et al. (2023) identified four dimensions: self-related, other-related, collective-related, and responsible decision-making competence. Although frameworks vary in their organization, the OECD framework emphasizes self-regulation, social understanding, cooperation, and deliberate action (OECD, 2021). These capacities remain relevant beyond school-based social and emotional learning because university study requires students to interpret feedback, work with peers, manage relationships in learning communities, and make choices about effort and priorities. Existing Chinese university studies have associated SEC with learning communities, school climate, and learning engagement (Chen C. et al., 2024; Chen Z. et al., 2024; Yao and Chen, 2023). Qualitative evidence from first-year students also shows that SEC development can occur through university-linked social practice that broadens perspectives, strengthens social connections, and supports responsible decision-making (Lyu et al., 2025). These university applications extend the general evidence that school-based social and emotional learning is associated with later behavioral and academic outcomes (Durlak et al., 2011; Taylor et al., 2017), while placing SEC within the more autonomous and socially varied context of higher education.

The possible relationship between SEC and academic resilience can first be understood through individual and self-regulatory resources. Self-awareness may help students recognize their emotional and learning responses, while emotion regulation and persistence may help them continue working when progress is slow. Responsible decision-making can support choices about when to revise a plan, seek assistance, or allocate effort, and goal maintenance can preserve direction across a period of academic difficulty. These processes overlap with the self-regulatory demands of resilient academic functioning without making SEC and resilience interchangeable. Social cognitive theory provides a broader basis for this account by emphasizing forethought, self-regulation, and self-reflection within the social contexts in which individuals act (Bandura, 2001). Self-regulated learning requires students to monitor progress, select strategies, and adjust effort; SEC may support the emotional and decision-related capacities through which those learning processes are enacted. The Study Demands-Resources perspective similarly treats personal and study resources as supports for academic resilience, engagement, and well-being (Bagdžiūnienė et al., 2025). Prospective associations of self-efficacy and motivation with later resilience further indicate that students’ beliefs about their capabilities and reasons for continued effort are relevant to adaptation over time (Yu et al., 2026).

A second route involves interpersonal and collective resources. University students respond to difficulty within social settings in which peers, teachers, and learning communities can provide information, feedback, encouragement, and practical support. Other-related competence may therefore be relevant to recognizing and using interpersonal resources, whereas collective-related competence concerns participation in and identification with groups. Social support has shown a prospective positive association with academic resilience among Chinese university students (Yu et al., 2026). At a broader level, a meta-analysis of postsecondary students found that belonging was positively related to motivation, self-efficacy, engagement, retention, and persistence intentions (Yang et al., 2026). Evidence that social practice can strengthen freshmen’s social connections and SEC also illustrates how relational learning experiences may develop during the university transition (Lyu et al., 2025). Together, this literature suggests that resilient academic functioning may draw on both personal regulation and students’ connections with others.

Research has nevertheless tended to examine these resources at an aggregate level. Emotional intelligence and other psychological resources have been associated with resilience, academic stress, and performance among university students (Abdelrahman et al., 2025; Ononye et al., 2022; Trigueros et al., 2020), and recent studies have clarified the roles of self-efficacy, motivation, social support, and study resources (Bagdžiūnienė et al., 2025; Yu et al., 2026). Such findings establish that social, emotional, and personal resources are relevant to resilience, but they do not show whether the relationship is distributed evenly across the dimensions of each construct. Both SEC and academic resilience are multidimensional. A total-score correlation cannot identify which SEC facets show conditional associations with which aspects of resilience after the remaining dimensions are considered, nor can it show which SEC dimensions carry more of the observed cross-construct connectivity. Aggregate approaches may therefore obscure theoretically meaningful heterogeneity in the relationship.

Network analysis provides a way to examine that heterogeneity. In a psychological network, nodes represent the dimensions under study and edges represent conditional associations after the other nodes in the network are taken into account (Cai et al., 2020; Epskamp and Fried, 2018; Epskamp et al., 2018a). Whereas bivariate correlations describe associations without adjustment for the remaining dimensions, both multiple regression and partial-correlation networks can estimate adjusted relationships. Partial-correlation networks are statistically related to node-wise regressions (Epskamp and Fried, 2018). Their value for the present questions lies in organizing the conditional associations among all ten dimensions within a common model, allowing the connections within and between the two constructs to be examined together without designating a single outcome. Strength describes a node’s overall absolute connectivity, whereas bridge strength summarizes its connections with nodes in another theoretically defined module (Jones et al., 2021). This distinction allows us to examine whether a dimension’s overall connectivity primarily reflects connections within its own construct or connections with dimensions of the other construct. Because the present questions concerned theoretically defined facets of SEC and academic resilience, a dimension-level network was used rather than an item-level network. This level of analysis retains conceptually interpretable constructs while reducing redundancy among closely related items within the same subscale. Network methods have increasingly been applied to university populations to examine how motivational, self-regulatory, professional, and resilience-related dimensions are organized and connected (Liu et al., 2024; Wang et al., 2025; Xu et al., 2026). Their application to SEC and academic resilience can therefore show whether conditional cross-construct associations are broadly distributed across dimensions or limited to specific dimension pairs.

The present study examined the dimension-level organization of SEC and academic resilience among students from six universities in Anhui Province, China. The analysis focused on the pattern of conditional associations among four SEC and six academic-resilience dimensions, the relative magnitude of retained cross-construct edges, and the SEC dimensions showing greater connectivity with academic-resilience dimensions. The theoretical aim was to refine the broad association between SEC and academic resilience by identifying how self-related, interpersonal, collective, and decision-related capacities connect with particular resilience facets. Combining aggregate and dimension-level evidence could thereby generate more specific propositions about the resources associated with academic adaptation. The following exploratory research questions guided the analysis:

*RQ1*: What is the dimension-level network structure of social and emotional competence and academic resilience?
*RQ2*: Which cross-construct edges show relatively large conditional associations?
*RQ3*: Which dimensions show relatively high overall strength, and which SEC dimensions show relatively high bridge strength with the academic-resilience dimensions?

## 2 Materials and methods

This cross-sectional survey examined dimension-level associations between social and emotional competence and academic resilience among university students.

### 2.1 Participants and procedure

Between 12 and 26 May 2026, a cross-sectional online questionnaire survey was conducted among university students from six universities in Anhui Province, China, using convenience sampling. The questionnaire was administered through the Wenjuanxing online survey platform and was distributed by the researchers or relevant teachers through class-based and course-based online communication groups. Participants were eligible for inclusion if they were aged 18 years or older, were currently enrolled in a regular higher education institution, were able to complete the questionnaire independently, and voluntarily agreed to participate in the study. University students were the target population because the study concerned social and emotional resources associated with academic adaptation in higher education, where students face increasing responsibility for organizing their learning and responding to academic challenges. Both questionnaires were developed for Chinese university students, supporting their selection for this population. The adult eligibility criterion was consistent with the electronic informed-consent procedure, while independent questionnaire completion was relevant to the self-report format.

The first questionnaire page described the study purpose, anonymous participation, and intended use of the data. Participants continued only after confirming that they were at least 18 years old and providing electronic informed consent. The study was approved by the Research Ethics Committee of Anqing Normal University (protocol code AQNU2026075; 18 March 2026). Of 1,210 submitted responses, 19 were excluded during data-quality screening. Responses were screened using two criteria: (a) a total questionnaire completion time of less than 180 s, with the threshold determined based on completion times observed during pilot testing; and (b) selection of the same response option for at least 10 consecutive items within either scale. Response patterns were evaluated before reverse scoring. Responses meeting either criterion were excluded. Of the 19 excluded responses, 11 met only the completion-time criterion, 6 met only the identical-response criterion, and 2 met both criteria. The final analytic sample comprised 1,191 participants, representing 98.43% of submitted responses. All questionnaire items were required, so the analytic dataset contained no item-level missing data. All eligible responses collected during the study period were included in the analysis.

Participants ranged in age from 18 to 24 years, with a mean age of 19.81 years (SD = 1.28). The sample included 526 male and 665 female students. Regarding year of study, 334 participants were first-year students, 322 were second-year students, 276 were third-year students, 228 were fourth-year students, and 31 were in their fifth year or above. Participants represented a range of academic disciplines, including science and engineering, humanities and social sciences, medicine, arts and physical education, agriculture and forestry, and other fields. Additional demographic variables included place of origin, only-child status, whether the participant held a student leadership position, self-reported family economic status, and recent experience of course failure or retaking during the past academic year. Detailed demographic characteristics are presented in Table 1. The six universities constituted a regional convenience sample; the inclusion of students from different years and disciplines provided variation within this sample but did not establish representativeness of Chinese university students.

**Table 1:** Demographic characteristics of the study sample (*N* = 1,191).

Variable	Category	*n*	%
Sex	Male	526	44.16
	Female	665	55.84
Year of study	Year 1	334	28.04
	Year 2	322	27.04
	Year 3	276	23.17
	Year 4	228	19.14
	Year 5 or above	31	2.60
Field of study	Science and engineering	480	40.30
	Humanities and social sciences	356	29.89
	Medicine	167	14.02
	Arts and physical education	83	6.97
	Agriculture and forestry	65	5.46
	Other	40	3.36
University source	University 01	219	18.39
	University 02	223	18.72
	University 03	219	18.39
	University 04	199	16.71
	University 05	183	15.37
	University 06	148	12.43
Place of origin	Urban	661	55.50
	Rural	530	44.50
Only-child status	No	668	56.09
	Yes	523	43.91
Student leadership position	No	849	71.28
	Yes	342	28.72
Self-reported family economic status	Financially disadvantaged	135	11.34
	Average	680	57.09
	Relatively good	283	23.76
	Affluent	93	7.81
Recent course failure or retaking	No	969	81.36
	Yes	222	18.64

Percentages are based on the full analytic sample. Age was 19.81 (SD = 1.28) years, with a range of 18–24 years. There were no missing values for the variables presented in the table. University identifiers are anonymised.

### 2.2 Measures

Both instruments were developed for Chinese university students and were administered in Chinese. Their measurement properties were evaluated using evidence from the original development studies and analyses of the present sample. The following subsections describe the instruments, response formats, scoring procedures, and original validation evidence. Section 2.5 details the current-sample evaluation of internal structure, reliability, and associations with related dimensions and an external academic criterion.

#### 2.2.1 Social and emotional competence

We used the University Student Social and Emotional Competence Scale developed by Chen et al. (2023). The scale contains 26 items across four dimensions: self-related competence (items 1–6), other-related competence (items 7–11), collective-related competence (items 12–19), and responsible decision-making competence (items 20–26). An example item is “I know my strengths and weaknesses.” Items are rated on a 5-point Likert scale from 1 (completely inconsistent with me) to 5 (completely consistent with me). No items are reverse scored. Dimension scores are the means of their constituent items, with higher scores indicating stronger competence. The original validation study supported the four-factor structure (CFI = 0.95, TLI = 0.94, RMSEA = 0.07) and reported Cronbach’s α of 0.97 for the total scale. In the present sample, the total-scale Cronbach’s *α* was 0.87, with dimension-level reliability reported in Table 2.

**Table 2:** Descriptive statistics, reliability, and standardized factor loading ranges (*N* = 1,191).

Variable	M	SD	Skewness	Kurtosis	Cronbach’s *α*	McDonald’s *ω*	Standardized loading range
Social and emotional competence total	3.24	0.56	−0.12	−0.31	0.87	0.89	—
Self-related competence	3.18	0.86	0.03	−0.71	0.84	0.87	0.60–0.86
Other-related competence	3.27	0.83	−0.18	−0.51	0.79	0.82	0.59–0.81
Collective-related competence	3.24	0.80	−0.18	−0.48	0.85	0.87	0.60–0.75
Responsible decision-making competence	3.28	0.79	−0.24	−0.43	0.84	0.86	0.65–0.82
Academic resilience total	3.74	0.64	−0.04	−0.40	0.83	0.84	—
Awareness	3.69	1.08	−0.14	−0.70	0.74	0.77	0.59–0.79
Acceptance	3.70	1.10	−0.10	−0.60	0.76	0.78	0.62–0.76
Goal orientation	3.68	1.07	0.08	−0.62	0.76	0.79	0.60–0.78
Meaning orientation	3.76	1.17	−0.15	−0.84	0.77	0.81	0.74–0.80
Present-moment focus	3.83	1.11	−0.22	−0.64	0.76	0.79	0.65–0.81
Cognitive defusion	3.78	1.00	−0.13	−0.57	0.79	0.82	0.54–0.81

M, mean; SD, standard deviation. Cronbach’s *α* and McDonald’s *ω* are internal-consistency coefficients. The social and emotional competence scale uses 5-point scoring, whereas the academic resilience questionnaire uses 6-point scoring. Standardized loading ranges refer to items within each dimension in the joint ten-factor WLSMV model. Dashes indicate that loading ranges are not applicable to the total-score rows. Item-level standardized loadings are reported in Supplementary Table S6. All estimates are based on *N* = 1,191.

#### 2.2.2 Academic resilience

We used the 24-item University Student Academic Resilience Questionnaire developed by Kong (2018). The instrument was developed from acceptance and commitment therapy and a resilience process framework, with item content informed by interviews, an open-ended questionnaire, and expert review. An initial pool of 35 items was reduced to 24 through item analysis and exploratory analysis (Kong, 2018). It comprises awareness, acceptance, goal orientation, meaning orientation, present-moment focus, and cognitive defusion. A representative awareness item is “I am clear about the necessity of receiving a university education.” Items are rated from 1 (completely inconsistent with me) to 6 (completely consistent with me). Negatively worded items (Items 16–24) were reverse-scored as 7 minus the original response before score calculation. Dimension scores were calculated as the means of their constituent items, and the total score was the mean of all 24 items, with higher scores indicating higher levels of the corresponding academic resilience dimensions. The original study reported a total Cronbach’s alpha of 0.83 and subscale values of 0.67–0.84. Kong (2018) also reported positive correlations with the Connor–Davidson Resilience Scale (*r* = 0.302) and a four-item academic buoyancy scale (*r* = 0.310), both reported as *p* < 0.05. These findings provided initial evidence of associations with related resilience constructs in the original validation study. The total-scale alpha was 0.83 in the present sample, with dimension-level reliability reported in Table 2. Definitions and representative items appear in Supplementary Table S8.

Acceptance, present-moment focus, and cognitive defusion share a theoretical basis with psychological flexibility processes (Hayes et al., 2006). Their inclusion in this questionnaire reflects Kong’s (2018) account of adaptation to academic difficulty. Acceptance items address recovering from setbacks, examining their causes, and acknowledging learning limitations; present-moment focus concerns maintaining involvement in learning tasks; and cognitive defusion concerns reduced interference from repetitive thoughts about academic difficulties and failure. Together with awareness, goal orientation, and meaning orientation, these dimensions operationalize self-reported responses and regulatory tendencies relevant to continued academic engagement. The measure therefore combines perceived recovery from setbacks with learning-related processes that may support adaptation.

#### 2.2.3 Self-reported academic rank

As an external criterion, participants reported their approximate academic rank within their major during the previous academic year. Responses were coded from 1 (bottom 25%) to 4 (top 25%), with higher values indicating better academic standing. The 52 participants selecting “not sure” were treated as having missing rank information, leaving 1,139 participants for the criterion-related analyses. They remained included in the measurement and network analyses.

### 2.3 Network analysis

The primary analyses were conducted in R version 4.6.1. Continuous variables were summarized with means and standard deviations, and categorical variables with counts and percentages. Cronbach’s alpha, McDonald’s omega, and descriptive statistics were calculated with psych version 2.6.5.

The research questions concerned relations among theoretically defined facets rather than associations among individual questionnaire items. The network therefore comprised four social and emotional competence dimensions and six academic resilience dimensions, grouped as two theoretical modules. Using dimension scores also reduced redundancy among closely related items within each subscale while retaining nodes with direct conceptual meaning. A Gaussian graphical model was estimated from Spearman correlations using EBICglasso with gamma = 0.50 in qgraph version 1.10.1 and bootnet version 1.9.1 (Epskamp et al., 2012; Epskamp and Fried, 2018; Epskamp et al., 2018b; Foygel and Drton, 2010; Friedman et al., 2008). Spearman correlations were selected as a rank-based approach appropriate for dimension scores derived from ordinal Likert-type responses. The EBIC hyperparameter gamma = 0.50 provides a commonly used balance between model sparsity and edge retention in psychological network estimation. Regularization can shrink small estimated edges to zero. An absent edge therefore indicates that a conditional association was not retained under the selected model specification; it does not establish the absence of a bivariate association or a zero conditional association in the population (Epskamp and Fried, 2018). Strength was the sum of absolute edge weights connected to a node. Bridge strength was the sum of absolute connections to the other module and was calculated with network tools version 1.6.0 (Jones et al., 2021; Robinaugh et al., 2016). Because the modules differed in size, substantive bridge-strength comparisons focused on the four social and emotional competence dimensions.

### 2.4 Accuracy, stability, and sensitivity

We used 5,000 nonparametric bootstrap samples to examine edge-weight accuracy and conduct exploratory edge and centrality difference tests. Bootstrap confidence intervals were treated as accuracy estimates rather than formal significance tests. We used 5,000 case-dropping bootstrap samples to estimate CS coefficients for strength and bridge strength at a correlation threshold of 0.70. Sensitivity analyses considered alternative EBIC hyperparameter values (*γ* = 0.25, 0.75, and 1.00), Pearson correlations, covariate adjustment, leave-one-university-out models, deletion of two overlapping self-related competence items, and an unregularized partial-correlation network. The tuning-parameter and unregularized comparisons used the same 1,191 participants, ten dimension scores, and Spearman correlation matrix as the primary model. We compared signed edge weights, nonzero status, and absolute-weight rankings among all 24 cross-construct edges. The item-deletion analysis excluded self-related competence items 5 and 6 (SEC05 and SEC06), which assess task persistence and long-term goal setting and thus share content with goal orientation. Self-related competence was rescored using items 1–4, and the network was re-estimated to examine the sensitivity of the SRC-GO edge to these overlapping items. Full specifications and results are provided in the Supplementary materials.

### 2.5 Measurement evaluation

We evaluated the measurement structure using confirmatory factor analysis in lavaan version 0.7.2 (Rosseel, 2012). Questionnaire items were treated as ordered categorical variables and estimated using weighted least squares mean- and variance-adjusted estimation (WLSMV). We examined the four-factor SEC model, the six-factor academic resilience model, and a joint model comprising ten correlated factors. In the joint model, items loaded on their corresponding theoretical factors. Model fit was assessed using *χ*^2^, degrees of freedom, CFI, TLI, RMSEA with 90% confidence intervals, and SRMR. Standardized factor loadings, composite reliability (CR), average variance extracted (AVE), and heterotrait–monotrait ratios (HTMT) were also examined. To evaluate a plausible alternative to treating self-related competence and goal orientation as distinct dimensions, we compared the joint ten-factor model with a nine-factor model that combined their items into a single factor while retaining the other eight factors. This comparison addressed their shared goal-setting and persistence content. Both models used the same 50 items and 1,191 participants and were compared using the WLSMV scaled chi-square difference test. Criterion-related evidence was examined using Spearman correlations between academic resilience scores and self-reported academic rank. The total-score association was the primary analysis, and the six dimension-level associations were secondary analyses. We calculated 95% percentile confidence intervals using 5,000 paired-case bootstrap samples and applied the Benjamini–Hochberg correction to the six secondary *p* values. Tests were two-sided, with asymptotic *p* values used because of tied ranks. These additional analyses were conducted in Python 3.12.14 using SciPy 1.18.1.

## 3 Results

### 3.1 Sample characteristics and descriptive statistics

The demographic characteristics of the participants are shown in Table 1.

The mean total score was 3.24 (SD = 0.56) for social and emotional competence and 3.74 (SD = 0.64) for academic resilience. At the aggregate level, the constructs showed a moderate positive association (Spearman’s rho = 0.41, *p* < 0.001). This total-score association provided an aggregate benchmark for the subsequent dimension-level network analysis. Across dimensions, Cronbach’s alpha ranged from 0.74 to 0.85 and McDonald’s omega from 0.77 to 0.87. The instruments use different response scales, so their raw means should not be compared directly (Table 2).

The four-factor SEC model yielded *χ*^2^(293) = 607.74, CFI = 0.96, TLI = 0.95, RMSEA = 0.04 (90% CI [0.03, 0.04]), and SRMR = 0.03, providing support for its proposed internal structure in the present sample. The joint ten-factor WLSMV model yielded *χ*^2^(1,130) = 1,927.42, CFI = 0.94, TLI = 0.93, RMSEA = 0.03 [90% CI (0.03, 0.03)], and SRMR = 0.03. Standardized item loadings ranged from 0.54 to 0.86, with dimension-specific ranges reported in Table 2. HTMT values ranged from 0.04 to 0.66 across the ten dimensions (Supplementary Table S1). The alternative nine-factor model combining self-related competence and goal orientation showed poorer fit: *χ*^2^(1,139) = 2,397.97, CFI = 0.91, TLI = 0.90, RMSEA = 0.04 [90% CI (0.04, 0.04)], and SRMR = 0.04. The WLSMV scaled chi-square difference test favored the ten-factor model, Δ*χ*^2^(9) = 117.46, *p* < 0.001. Together, these findings supported retaining the proposed dimensions, with the model comparison providing specific evidence for distinguishing self-related competence from goal orientation.

For academic resilience specifically, the six-factor WLSMV model yielded *χ*^2^(237) = 748.364, CFI = 0.934, TLI = 0.923, RMSEA = 0.047 [90% CI (0.043, 0.051)], and SRMR = 0.040 (Supplementary Table S9). These results provided support for the proposed internal structure, although AVE values were below 0.50 for awareness, acceptance, goal orientation, and cognitive defusion, indicating limitations in convergent evidence for these dimensions (Supplementary Table S10).

The academic resilience total score was positively associated with self-reported academic rank [*N* = 1,139, Spearman’s *ρ* = 0.294, 95% bootstrap CI (0.238, 0.348), *p* < 0.001]. Dimension-level correlations ranged from 0.096 for acceptance to 0.410 for goal orientation, and all six remained statistically significant after Benjamini–Hochberg correction (Supplementary Table S14). These findings provided additional criterion-related evidence based on self-reported academic standing.

### 3.2 Network structure and cross-construct edges

The ten-node network retained 28 of 45 possible edges, including 19 within-construct and nine cross-construct edges. Thus, retained connections were more frequent within the two constructs than between them, and the cross-construct edges were limited to a subset of the 24 possible SEC-academic-resilience pairs. Twenty-seven retained edges were positive and one was negative. The largest within-construct edges linked acceptance with cognitive defusion (0.24), other-related with collective-related competence (0.23), present-moment focus with cognitive defusion (0.22), and self-related with responsible decision-making competence (0.21). The complete bivariate correlation matrix is reported in Supplementary Table S7 (Figure 1).

**Figure 1:**
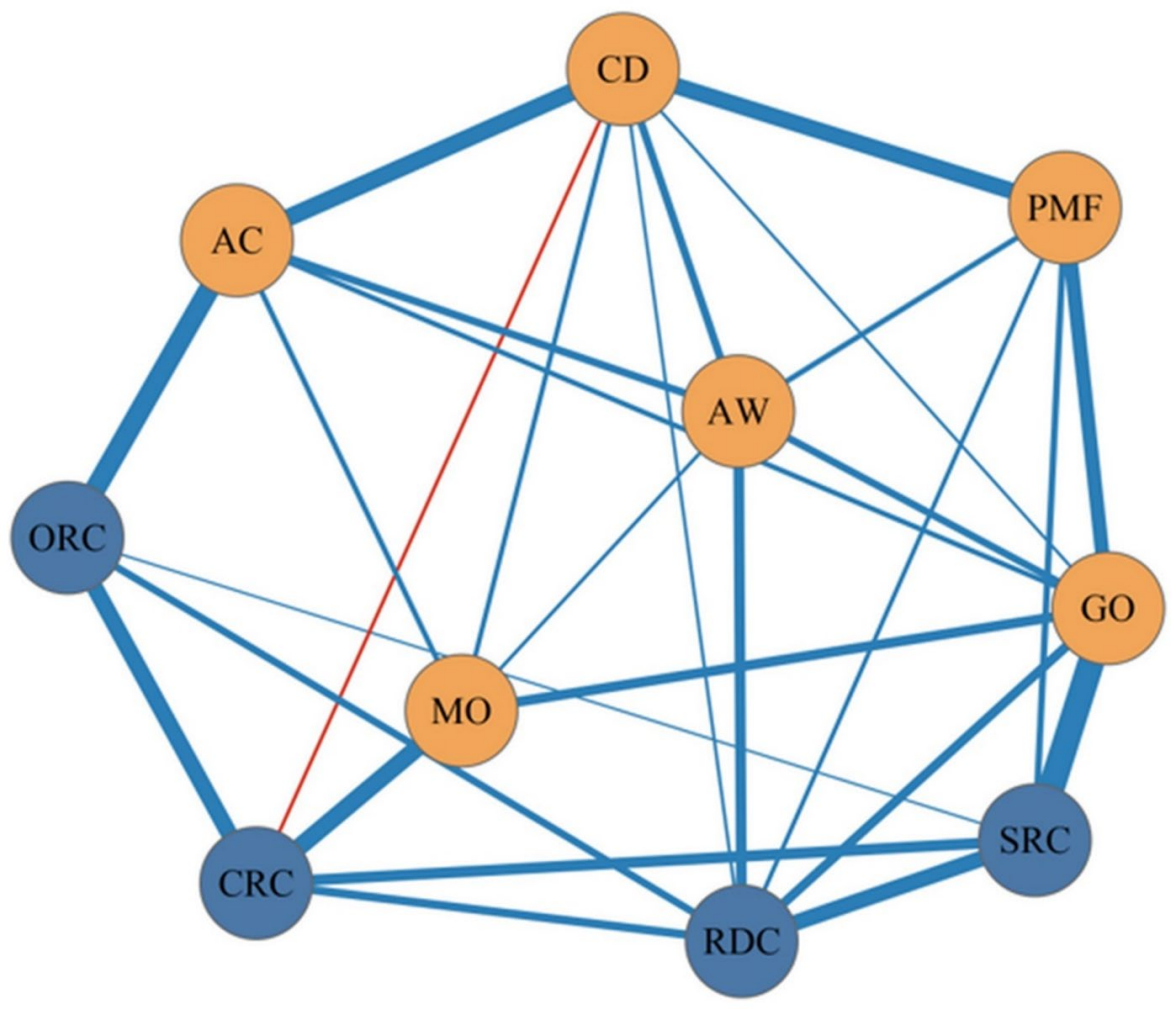
Network structure of social and emotional competence and academic resilience. Node (SRC, self-related competence; ORC, other-related competence; CRC, collective-related competence; RDC, responsible decision-making competence; AW, awareness; AC, acceptance; GO, goal orientation; MO, meaning orientation; PMF, present-moment focus; CD, cognitive defusion). Blue nodes represent social and emotional competence, and orange nodes represent academic resilience. Blue edges indicate positive partial associations and red edges negative partial associations. Edge width represents the absolute edge weight.

The largest cross-construct edge connected self-related competence with goal orientation (0.37). Other relatively large connections linked other-related competence with acceptance (0.29) and collective-related competence with meaning orientation (0.24). Smaller links involving responsible decision-making were observed with awareness (0.13) and goal orientation (0.11). The bootstrap comparison of the SRC-GO edge with the other retained cross-construct edges is reported in Supplementary Figure S2. Complete estimates are reported in Table 3.

**Table 3:** Cross-construct edge estimates and bootstrap accuracy results.

SEC dimension	AR dimension	Edge weight	Bootstrap 95% CI
Self-related competence	Goal orientation	0.37	[0.319, 0.416]
Other-related competence	Acceptance	0.29	[0.235, 0.334]
Collective-related competence	Meaning orientation	0.24	[0.185, 0.289]
Responsible decision-making competence	Awareness	0.13	[0.078, 0.185]
Responsible decision-making competence	Goal orientation	0.11	[0.057, 0.167]
Self-related competence	Present-moment focus	0.07	[0.017, 0.124]
Responsible decision-making competence	Present-moment focus	0.04	[0.000, 0.098]
Collective-related competence	Cognitive defusion	−0.02	[−0.089, 0.000]
Responsible decision-making competence	Cognitive defusion	0.02	[0.000, 0.071]

Bootstrap confidence intervals describe estimation accuracy. CI, confidence interval.

### 3.3 Node and bridge strength

Goal orientation had the highest strength value (0.95), followed by self-related competence (0.80), collective-related competence (0.73), acceptance (0.72), and responsible decision-making competence (0.70). Figure 2A shows the distribution across all nodes; exact values are reported in Supplementary Table S12.

**Figure 2:**
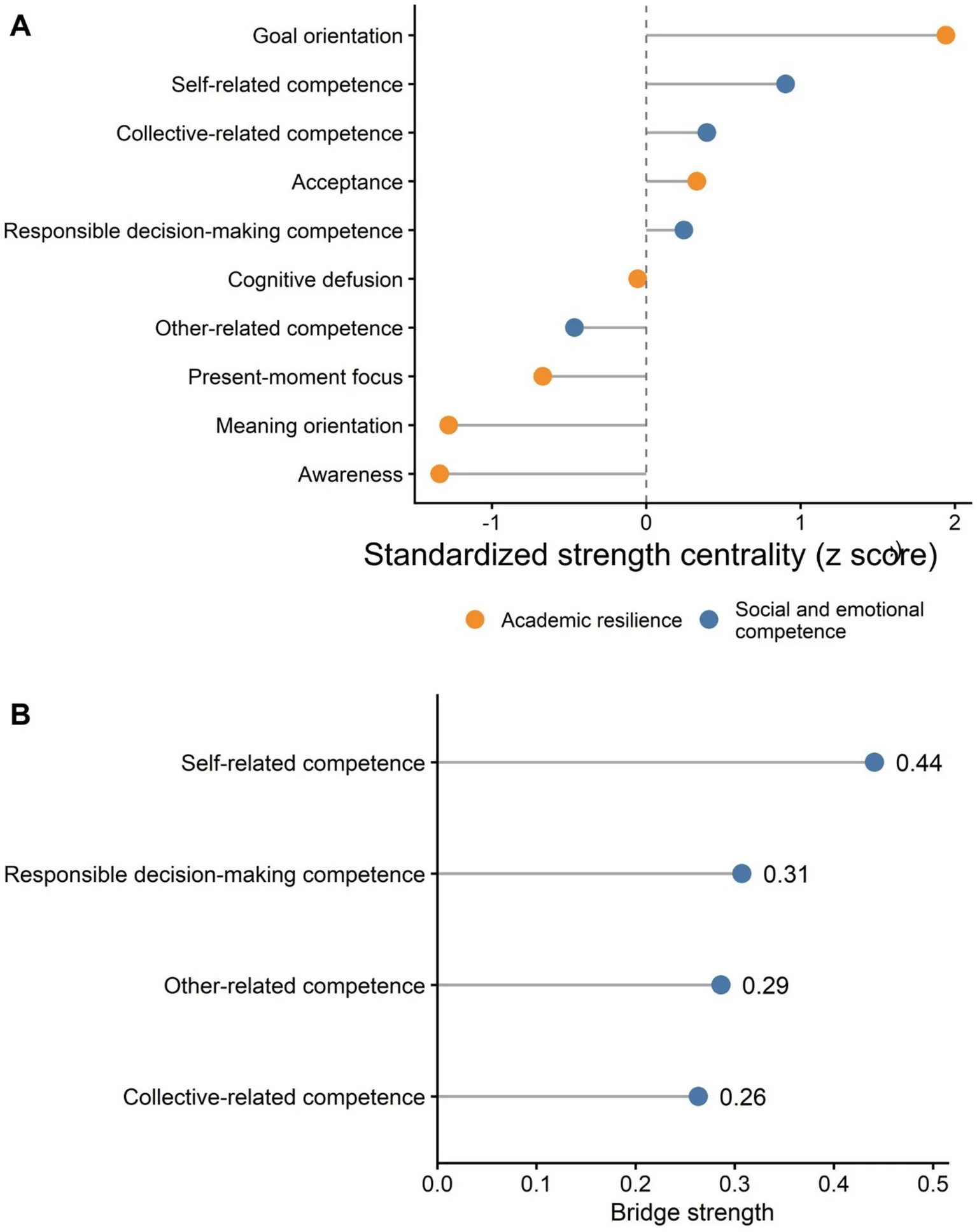
Node strength and SEC bridge strength. Panel **(A)** shows standardized strength centrality across all nodes. Panel **(B)** shows unstandardized bridge strength for the four social and emotional competence dimensions.

Within the social and emotional competence module, self-related competence had the highest bridge strength (0.44), followed by responsible decision-making competence (0.31), other-related competence (0.29), and collective-related competence (0.26). Figure 2B shows these estimates, and bootstrap comparisons are reported in Supplementary Figure S4. The rankings are reported descriptively as features of the estimated cross-sectional network.

### 3.4 Network stability and sensitivity

Strength and bridge strength showed high case-dropping stability (CS = 0.75). Across the four EBICglasso settings (*γ* = 0.25, 0.50, 0.75, and 1.00), the SRC–GO, ORC–AC, and CRC–MO edges remained positive, with weights ranging from 0.370 to 0.373, 0.286 to 0.291, and 0.238 to 0.243, respectively. Their corresponding unregularized partial correlations were 0.388, 0.317, and 0.280. These edges consistently ranked first, second, and third by absolute weight among the 24 cross-construct edges in all five networks. Goal orientation retained the highest strength, and self-related competence retained the highest bridge strength within the SEC module. The *γ* = 0.75 and 1.00 networks were identical to the primary network, while *γ* = 0.25 retained one additional cross-construct edge, ORC–PMF (−0.008). Edge-weight correlations with the primary network ranged from 0.995 to 1.000 across alternative γ values and were 0.968 for the unregularized network. Although all nine primary cross-construct edges remained nonzero and retained their signs, some weaker edges changed in magnitude or rank. Full comparisons are reported in Supplementary Tables S11, S15. Specifically, removing SEC05 and SEC06 reduced the SRC-GO edge from 0.37 to 0.32, although it remained the largest cross-construct edge in the re-estimated network. The four-item self-related competence score had a Cronbach’s *α* of 0.74 (Supplementary Tables S4, S11).

## 4 Discussion

### 4.1 Overall network pattern

The moderate total-score association between SEC and academic resilience (rho = 0.41) coexisted with a markedly uneven pattern of dimension-level connectivity. Aggregate scores showed that students who reported greater SEC also tended to report greater academic resilience. The network added a more specific account: only nine of the 24 possible cross-construct connections were retained. This count describes the selected regularized network; dimension pairs without retained edges may still have bivariate associations or conditional associations that were shrunk to zero. The three largest cross-construct edges were SRC-GO, ORC-Acceptance, and CRC-Meaning. The two constructs were therefore related without being uniformly connected across all of their dimensions. An overall association does not imply that every dimension of one construct will show a comparable conditional association with every dimension of the other.

The pattern extends earlier university research that has linked emotional intelligence, personal resources, and social resources with academic resilience, stress, engagement, and performance (Abdelrahman et al., 2025; Bagdžiūnienė et al., 2025; Ononye et al., 2022; Trigueros et al., 2020; Yu et al., 2026). Those studies establish the relevance of broader psychological resources. The present analysis identifies which dimension-level conditional associations remained after the other measured dimensions were taken into account. In particular, the largest retained links connected self-related competence with goal orientation, other-related competence with acceptance, and collective-related competence with meaning orientation. This conditional pattern complements the network reported between positive psychological capital and academic resilience among nursing undergraduates (Xu et al., 2026), while broadening the focus to a multidimensional SEC framework.

Within-construct edges were more frequent than cross-construct edges in the selected network. At the same time, the cross-construct links involved individual, interpersonal, collective, and decision-related facets. Their coexistence suggests that academic adaptation is not organized around self-regulation alone. Personal regulation may help students manage effort and maintain direction, whereas relational resources may shape how they use feedback, support, and membership in a learning community. The theoretical contribution is to give the differentiated SEC framework a more specific account of its connections with academic resilience: the most prominent cross-construct associations of self-related, other-related, and collective-related competence involved goal orientation, acceptance, and meaning orientation, respectively. This pattern suggests that the SEC facets have distinct profiles of association with resilience, which a total-score relationship does not reveal. It provides a basis for testing whether these particular pairings also characterize changes in students’ responses to academic difficulty over time.

### 4.2 Self-related competence and goal orientation

The largest cross-construct edge linked self-related competence with goal orientation. Self-related competence includes reflection on one’s capacities and emotions, regulation of emotional responses, persistence, and long-term goal setting. Goal orientation in the academic-resilience instrument concerns setting academic goals, planning, and sustaining effort toward those goals. From a social cognitive perspective, forethought gives action a future direction, while self-reflection and self-regulation support the evaluation and adjustment of ongoing behavior (Bandura, 2001). The SRC-GO association is consistent with this coordination: students’ understanding and regulation of themselves are closely connected with the academic goals around which effort is organized. This interpretation links the observed association to a broader account of purposeful action, with its temporal organization remaining a question for longitudinal research.

This interpretation is consistent with research on self-regulated learning in higher education. Goal-orientation patterns have been associated with university students’ use of self-regulated reading strategies (Kolić-Vehovec et al., 2008). During disrupted online learning, adaptive self-regulatory practices buffered the association between metacognitive challenges and academic performance, and a semester-long intervention reduced several forms of academic challenge among first-year undergraduates (Hadwin et al., 2022). Among Chinese university students learning English as a foreign language, effort regulation, time management, and metacognitive strategies were also related to lower procrastination or greater academic success (Tao et al., 2025). Meta-analytic evidence further indicates that self-regulated-learning programs can improve performance, strategy use, and motivation in university students (Theobald, 2021). The SRC-GO edge adds to this literature by locating the close connection between regulatory capacity and goal maintenance within a broader network that also includes interpersonal and collective facets of SEC.

Goal orientation also had the highest overall strength in the ten-node network. Its connectivity was not limited to self-related competence: it was linked with several academic-resilience dimensions and with responsible decision-making competence. Descriptively, this places goal pursuit near the center of the network’s organization. The pattern is compatible with accounts of academic resilience that emphasize persistence and adaptive engagement following setbacks (Cassidy, 2016; Martin and Marsh, 2006, 2008). Goals can connect students’ reasons for continuing with the planning and effort required to continue, which may explain why goal orientation showed broad connectivity across this particular set of dimensions.

Among the SEC dimensions, self-related competence had the highest bridge strength, primarily reflecting its strong connection with goal orientation. This indicates the largest sum of absolute cross-construct edge weights rather than connections with the greatest number of resilience dimensions, locating its prominence in a specific self-regulatory pairing. Shared goal-setting and persistence content may nevertheless contribute to this connection. The SRC-GO edge remained the largest cross-construct edge after SEC05 and SEC06 were removed, suggesting that its prominence was not wholly dependent on these two items. However, deletion also changed the content and reliability of the self-related competence score, so the lower edge estimate should not be attributed exclusively to reduced overlap. The sensitivity analysis supports the persistence of the association while leaving some conceptual overlap between the dimensions possible.

### 4.3 Other cross-construct connections

The connection between other-related competence and acceptance adds an interpersonal component to the network. Other-related competence concerns understanding and responding to other people, capacities that can influence how students interpret feedback and whether they recognize and use available support. Acceptance, as measured here, concerns recovering from academic setbacks, examining their causes, and acknowledging limitations in learning ability. The Study Demands-Resources perspective places these interpersonal processes within a learning environment that can provide feedback and support. Consistent with this perspective, Bagdžiūnienė et al. (2025) found that study resources, including feedback and support from peers and teaching staff, were positively associated with academic resilience. One possible interpretation of the ORC-acceptance edge is that understanding others and engaging with their perspectives may help students acknowledge learning limitations and consider feedback following difficulties. This proposed explanation concerns how interpersonal competence might relate to acceptance; the availability and use of support were not directly tested in the present network.

Collective-related competence was most strongly connected across constructs with meaning orientation. Collective competence concerns students’ relation to groups and shared activities, while meaning orientation concerns locating academic effort and difficulty within a broader sense of purpose. Their association may reflect the role of learning communities in giving academic work social significance. This interpretation is consistent with evidence that postsecondary belonging is related more strongly to motivation, self-efficacy, and engagement than to direct performance indicators, and is also associated with retention and persistence intentions (Yang et al., 2026). University-linked social practice has likewise been described as strengthening freshmen’s social connections, social awareness, confidence, and responsible decision-making (Lyu et al., 2025). The CRC-meaning edge thus broadens the paper’s central self-regulatory account by indicating that collective identification and purpose may form a second, relational strand of academic adaptation.

Smaller links involving responsible decision-making with awareness and goal orientation suggest that deliberate academic choices may also be related to reflective and goal-directed aspects of resilience, although these connections were weaker than the three leading cross-construct edges.

### 4.4 Implications for educational psychology

For educational-psychology research, the findings show why academic resilience and SEC may be more informative when their facets are retained. Total scores remain useful for describing whether students with greater social and emotional resources also tend to report greater resilience. A dimension-level perspective answers a different question by showing which dimensions remain conditionally associated after the remaining dimensions are taken into account. Social cognitive theory and the Study Demands-Resources perspective offer complementary interpretations of this configuration. The former explains the coordination of purposeful action and personal regulation within social contexts, while the latter directs attention to the personal and contextual resources relevant to academic adaptation (Bandura, 2001; Bagdžiūnienė et al., 2025). Together, they support examining self-regulatory, interpersonal, and collective capacities as differentiated resources whose associations with specific resilience facets require further investigation. Other-related and collective-related competence concern students’ capacities for relating to others and groups, while the availability of external support was not directly assessed. These findings therefore offer an exploratory refinement of the resource perspective at the level of personal competencies.

The network also provides focused hypotheses for longitudinal work. Studies with repeated measurements could test whether change in self-related competence precedes, follows, or develops jointly with change in goal orientation. Parallel analyses could examine temporal relations between other-related competence and acceptance and between collective-related competence and meaning orientation. These associations may not remain constant across a degree program. Transition into university, assessment periods, course failure, and preparation for graduation may alter the balance between personal regulation and relational support. Modeling networks across such periods would show whether the present cross-sectional organization is stable or context dependent. Distinguishing within-person change from between-person differences would also establish whether the associations observed across students describe how academic adaptation unfolds within individual students.

The findings offer a restrained educational implication. Research and student-support practice may benefit from considering both self-regulatory and relational resources when examining responses to academic difficulty. Support for planning and persistence addresses only part of the pattern; students’ use of feedback, social connection, and sense of purpose may also be relevant. The immediate value of the present results lies in refining assessment and generating prospective questions that can be evaluated across changing educational contexts.

### 4.5 Limitations

The sampling design limits the population to which the estimates can be generalized. Participants were recruited by convenience from six universities in Anhui Province, and the sample was not designed to represent all institutions, regions, disciplines, or types of higher education in China. Institutional practices and local labor-market or educational conditions may shape both SEC and responses to academic difficulty. Replication in more diverse regions and institutions is needed before the observed network pattern is treated as characteristic of Chinese university students more broadly.

The cross-sectional, self-report design sets a second boundary. Conditional associations do not establish temporal ordering, and the present data cannot show whether SEC dimensions precede changes in resilience, arise alongside them, or reflect reciprocal development. Measuring both constructs in the same questionnaire may also introduce common-source variance. Repeated assessments across periods of changing academic demand, complemented where possible by behavioral, teacher, or peer indicators, would provide a stronger basis for examining temporal organization and contextual change.

Measurement also affects the interpretation of the network. Self-related competence and goal orientation share some goal-setting and persistence content, so conceptual overlap may have increased the SRC-GO edge. Several dimensions had AVE values below 0.50, and the network was estimated from observed dimension scores rather than latent variables; measurement error may therefore have influenced edge magnitudes. The academic resilience questionnaire combines perceived responses to setbacks with regulatory tendencies relevant to learning, and its acceptance, present-moment focus, and cognitive defusion dimensions overlap conceptually with psychological flexibility. Because psychological flexibility was not assessed independently, the present analyses cannot establish discriminant validity between these constructs. The additional association with academic rank provides limited criterion-related evidence: rank was a coarse, retrospective self-report indicator collected in the same survey, and it does not establish prospective prediction or recovery following adversity. Future studies should assess psychological flexibility alongside alternative academic resilience measures and follow students across identifiable academic setbacks to examine construct distinctiveness and adaptation over time.

## 5 Conclusion

This study found a moderate aggregate association between SEC and academic resilience alongside a selective pattern of dimension-level cross-construct connectivity. Self-related competence and goal orientation formed the strongest cross-construct connection; goal orientation had the highest overall strength, and self-related competence showed the greatest connectivity with academic-resilience dimensions among the SEC facets. Other-related competence with acceptance and collective-related competence with meaning orientation added a relational strand to the predominantly self-regulatory pattern. The findings therefore refine aggregate-score evidence by showing which aspects of personal regulation and social connection were conditionally related in this sample. Longitudinal research across periods of transition and academic stress is needed to establish how these connections develop and whether their organization changes with educational context.

## Data Availability

The raw data supporting the conclusions of this article will be made available by the authors, without undue reservation.
