# Supplementary material for: Self-related competence and goal orientation form the strongest cross-construct connection between social and emotional competence and academic resilience in university students: a dimension-level network analysis
Source: Front Psychol. 2026 Oct 2;17:1966171. doi: 10.3389/fpsyg.2026.1966171 (PMC13672553; doi:10.3389/fpsyg.2026.1966171)
Supplement: Supplementary file 1 [file Supplementary_file_1.docx]

**Supplementary Material**

**Contents**

Supplementary Table S1. Heterotrait-monotrait ratio matrix

Supplementary Table S2. Comparison of the ten-factor and nine-factor measurement models

Supplementary Table S3. Leave-one-university-out sensitivity analysis

Supplementary Table S4. Item-deletion sensitivity analysis for self-related competence

Supplementary Table S5. Academic resilience node bridge-strength estimates

Supplementary Table S6. Standardized factor loadings for all items in the ten-factor confirmatory factor analysis

Supplementary Table S7. Spearman correlation matrix of the ten network dimensions

Supplementary Table S8. Definitions and representative items of the six academic resilience dimensions

Supplementary Table S9. Confirmatory factor analysis fit indices

Supplementary Table S10. Composite reliability, average variance extracted, and standardized loading ranges

Supplementary Table S11. Sensitivity analyses

Supplementary Table S12. Node strength and bridge strength

Supplementary Table S13. Nonzero retention of cross-construct edges

Supplementary Table S14. Associations between academic resilience scores and self-reported academic rank

Supplementary Table S15. Cross-construct edge weights and absolute-weight ranks across network specifications

Supplementary Figure S1. Partial-correlation matrix of the ten network dimensions

Supplementary Figure S2. Bootstrapped difference test among cross-construct edge weights

Supplementary Figure S3. Bootstrapped strength-centrality difference test

Supplementary Figure S4. Bootstrapped bridge-strength difference test

Supplementary Figure S5. Case-dropping bootstrap stability of strength and bridge strength

**Supplementary Methods**

**Measurement checks**

The internal structure of the measures was examined using confirmatory factor analysis in lavaan version 0.7.2. Ordered categorical items were estimated with WLSMV. We report chi-square, degrees of freedom, CFI, TLI, RMSEA with 90% confidence intervals, and SRMR. CR, AVE, and HTMT were calculated with semTools version 0.5.9. The joint ten-factor model was compared with a nine-factor alternative in which all self-related competence and goal orientation items loaded on a single factor, with the remaining eight factors retained. This comparison addressed the shared goal-setting and persistence content of the two dimensions. Both models used the same 50 items and 1,191 participants and were compared using the WLSMV scaled chi-square difference test.

**Sensitivity analyses**

Sensitivity analyses used gamma values of 0.25, 0.75, and 1.00; Pearson correlations; covariate-adjusted residuals; leave-one-university-out models; deletion of items 5 and 6 from self-related competence; and an unregularized Spearman partial-correlation network. The covariate-adjusted model included age, sex, year of study, field of study, and university source. For the tuning-parameter comparisons, γ was varied across 0.25, 0.50, 0.75, and 1.00 while the sample, Spearman correlation matrix, and remaining estimation settings were held constant. The unregularized network was obtained by inverting the same Spearman correlation matrix and converting the resulting precision matrix into partial correlations. No penalization or edge selection was applied. Overall similarity was assessed using Spearman correlations between the 45 corresponding signed off-diagonal edge weights, including zeros. We additionally compared signed weights, nonzero status, and absolute-weight ranks across all 24 cross-construct edges. Nonzero status and ranks were determined from unrounded estimates, with average ranks used for ties. The item-deletion analysis targeted SEC05 and SEC06 because their task-persistence and long-term-goal-setting content overlaps with the goal-setting and sustained-effort content of goal orientation. The revised self-related competence score was calculated as the mean of SEC01–SEC04. The remaining dimension scores were retained, and the ten-node network was re-estimated using the primary Spearman-based EBICglasso specification with gamma = 0.50.

**Supplementary Results**

**Measurement checks**

The four-factor social and emotional competence model, six-factor academic-resilience model, and joint ten-factor model showed acceptable fit (Supplementary Table S9). Across the ten dimensions, CR ranged from 0.77 to 0.87, AVE from 0.44 to 0.58, and standardized loadings from 0.54 to 0.86 (Supplementary Tables S6 and S10). HTMT values ranged from 0.04 to 0.66 and remained below 0.85 (Supplementary Table S1). The scaled chi-square difference test favored the ten-factor model over the merged nine-factor model, delta chi-square = 117.46, delta df = 9, p < 0.001 (Supplementary Table S2). Seven dimensions had AVE values below 0.50, although their CR values exceeded 0.70.

**Sensitivity analyses**

The three largest cross-construct edges, SRC–GO, ORC–AC, and CRC–MO, remained positive and ranked first, second, and third, respectively, across all five model specifications (Supplementary Table S15). Across the four regularized networks, their weights ranged from 0.3704 to 0.3731, 0.2861 to 0.2909, and 0.2383 to 0.2427; the corresponding unregularized estimates were 0.3878, 0.3165, and 0.2803. The γ = 0.50, 0.75, and 1.00 settings selected the same candidate model from the graphical-lasso path and therefore produced identical networks. The γ = 0.25 network retained 29 edges, including ten cross-construct edges, compared with 28 and nine in the primary network. Correlations between the 45 edge weights and the primary network were 0.995 for γ = 0.25, 1.000 for γ = 0.75 and 1.00, and 0.968 for the unregularized network (Supplementary Table S11). All nine primary cross-construct edges remained nonzero and retained their signs across the five networks. Weaker connections showed greater variation: ORC–PMF was zero in the primary model but was estimated at −0.0079 under γ = 0.25 and −0.0597 without regularization. CRC–CD changed from −0.0249 in the primary model to −0.0815 without regularization. In the unregularized network, CRC–CD moved from eighth to seventh in absolute cross-construct rank, RDC–PMF from seventh to ninth, and RDC–CD from ninth to eleventh. Goal orientation and self-related competence retained the highest strength and highest SEC bridge strength, respectively, across all five networks. Leave-one-university-out models produced correlations of 0.964-0.991 (Supplementary Table S3). Removing SEC05 and SEC06 reduced the SRC-GO edge from 0.37 to 0.32; the four-item SRC score had a Cronbach’s α of 0.74 (Supplementary Table S4). SRC-GO remained the largest cross-construct edge, GO retained the highest strength point estimate, and SRC retained the highest bridge-strength point estimate within the SEC module (Supplementary Table S11).

**Supplementary Table S1.** Heterotrait-monotrait ratio matrix

| **Node** | **SRC** | **ORC** | **CRC** | **RDC** | **AW** | **AC** | **GO** | **MO** | **PMF** | **CD** |
| --- | --- | --- | --- | --- | --- | --- | --- | --- | --- | --- |
| SRC | - | 0.20 | 0.32 | 0.46 | 0.17 | 0.15 | 0.66 | 0.20 | 0.34 | 0.14 |
| ORC | 0.20 | - | 0.39 | 0.27 | 0.12 | 0.48 | 0.18 | 0.14 | 0.04 | 0.16 |
| CRC | 0.32 | 0.39 | - | 0.29 | 0.05 | 0.12 | 0.26 | 0.42 | 0.06 | 0.04 |
| RDC | 0.46 | 0.27 | 0.29 | - | 0.31 | 0.12 | 0.41 | 0.16 | 0.26 | 0.15 |
| AW | 0.17 | 0.12 | 0.05 | 0.31 | - | 0.25 | 0.31 | 0.18 | 0.24 | 0.29 |
| AC | 0.15 | 0.48 | 0.12 | 0.12 | 0.25 | - | 0.24 | 0.22 | 0.15 | 0.43 |
| GO | 0.66 | 0.18 | 0.26 | 0.41 | 0.31 | 0.24 | - | 0.32 | 0.46 | 0.23 |
| MO | 0.20 | 0.14 | 0.42 | 0.16 | 0.18 | 0.22 | 0.32 | - | 0.14 | 0.15 |
| PMF | 0.34 | 0.04 | 0.06 | 0.26 | 0.24 | 0.15 | 0.46 | 0.14 | - | 0.38 |
| CD | 0.14 | 0.16 | 0.04 | 0.15 | 0.29 | 0.43 | 0.23 | 0.15 | 0.38 | - |

*Note.* The maximum HTMT value was 0.66 between self-related competence and goal orientation; all values were below 0.85. SRC = self-related competence; ORC = other-related competence; CRC = collective-related competence; RDC = responsible decision-making competence; AW = awareness; AC = acceptance; GO = goal orientation; MO = meaning orientation; PMF = present-moment focus; CD = cognitive defusion.

**Supplementary Table S2.** Comparison of the ten-factor and nine-factor measurement models

| **Comparison** | **Δχ²** | **Δdf** | **p** | **ΔCFI** | **ΔRMSEA** | **Interpretation** |
| --- | --- | --- | --- | --- | --- | --- |
| Joint ten-factor versus nine-factor model | 117.46 | 9 | <0.001 | 0.03 | 0.01 | Ten-factor model preferred |

*Note.* The comparison used the WLSMV scaled chi-square difference test. Positive ΔCFI indicates better fit for the ten-factor model; ΔRMSEA is calculated as nine-factor minus ten-factor RMSEA.

**Supplementary Table S3.** Leave-one-university-out sensitivity analysis

| **University omitted** | **Remaining N** | **Nonzero edges** | **Largest cross-construct edge** | **Edge weight** | **Highest strength node** | **Highest bridge-strength SEC node** | **Edge-weight correlation** |
| --- | --- | --- | --- | --- | --- | --- | --- |
| University 01 | 972 | 28 | SRC-GO | 0.33 | GO | SRC | 0.991 |
| University 02 | 968 | 29 | SRC-GO | 0.38 | GO | SRC | 0.976 |
| University 03 | 972 | 27 | SRC-GO | 0.38 | GO | SRC | 0.978 |
| University 04 | 992 | 32 | SRC-GO | 0.37 | GO | SRC | 0.964 |
| University 05 | 1008 | 30 | SRC-GO | 0.38 | GO | SRC | 0.987 |
| University 06 | 1043 | 30 | SRC-GO | 0.39 | GO | SRC | 0.985 |

*Note.* Edge-weight correlation is Spearman's rank correlation between the vectors of the 45 corresponding edge-weight estimates from each leave-one-university-out network and the primary network. SRC = self-related competence; ORC = other-related competence; CRC = collective-related competence; RDC = responsible decision-making competence; GO = goal orientation. In all six analyses, SRC-GO remained the largest cross-construct edge, GO retained the highest strength point estimate, and SRC retained the highest bridge-strength point estimate within the social and emotional competence module. The ordering of the remaining SEC bridge-strength estimates varied across omitted universities.

**Supplementary Table S4.** Item-deletion sensitivity analysis for self-related competence

| **Model** | **Items retained for self-related competence** | **Cronbach's α** | **SRC-GO edge weight** |
| --- | --- | --- | --- |
| Primary network | SEC01–SEC06 | 0.84 | 0.37 |
| Item-deletion network | SEC01–SEC04 | 0.74 | 0.32 |

*Note.* SRC = self-related competence; GO = goal orientation. SEC05 and SEC06 assess task persistence and long-term goal setting, which overlap conceptually with goal orientation. In the item-deletion network, SRC was calculated as the mean of SEC01–SEC04. Both networks used Spearman correlations and EBICglasso with gamma = 0.50. SRC-GO remained the largest cross-construct edge after item deletion (Supplementary Table S11). Edge weights are reported to two decimal places.

**Supplementary Table S5.** Academic resilience node bridge-strength estimates from the primary EBICglasso network

| **Dimension** | **Bridge strength** |
| --- | --- |
| Awareness | 0.13 |
| Acceptance | 0.29 |
| Goal orientation | 0.48 |
| Meaning orientation | 0.24 |
| Present-moment focus | 0.11 |
| Cognitive defusion | 0.05 |

*Note.* Values are unstandardized bridge-strength estimates from the primary EBICglasso network. Because the social and emotional competence and academic resilience modules contained different numbers of nodes, these academic resilience values are provided for descriptive purposes only and were not compared substantively with values in the social and emotional competence module.

**Supplementary Table S6. Standardized factor loadings for all items in the ten-factor confirmatory factor analysis**

| **Construct** | **Dimension** | **Item** | **Standardized loading** |
| --- | --- | --- | --- |
| SEC | Self-related competence | SEC01 | 0.60 |
| SEC | Self-related competence | SEC02 | 0.68 |
| SEC | Self-related competence | SEC03 | 0.70 |
| SEC | Self-related competence | SEC04 | 0.68 |
| SEC | Self-related competence | SEC05 | 0.81 |
| SEC | Self-related competence | SEC06 | 0.86 |
| SEC | Other-related competence | SEC07 | 0.67 |
| SEC | Other-related competence | SEC08 | 0.69 |
| SEC | Other-related competence | SEC09 | 0.81 |
| SEC | Other-related competence | SEC10 | 0.59 |
| SEC | Other-related competence | SEC11 | 0.66 |
| SEC | Collective-related competence | SEC12 | 0.71 |
| SEC | Collective-related competence | SEC13 | 0.70 |
| SEC | Collective-related competence | SEC14 | 0.75 |
| SEC | Collective-related competence | SEC15 | 0.68 |
| SEC | Collective-related competence | SEC16 | 0.69 |
| SEC | Collective-related competence | SEC17 | 0.60 |
| SEC | Collective-related competence | SEC18 | 0.68 |
| SEC | Collective-related competence | SEC19 | 0.65 |
| SEC | Responsible decision-making competence | SEC20 | 0.66 |
| SEC | Responsible decision-making competence | SEC21 | 0.82 |
| SEC | Responsible decision-making competence | SEC22 | 0.72 |
| SEC | Responsible decision-making competence | SEC23 | 0.67 |
| SEC | Responsible decision-making competence | SEC24 | 0.65 |
| SEC | Responsible decision-making competence | SEC25 | 0.66 |
| SEC | Responsible decision-making competence | SEC26 | 0.66 |
| AR | Awareness | AR01 | 0.59 |
| AR | Awareness | AR02 | 0.79 |
| AR | Awareness | AR03 | 0.66 |
| AR | Awareness | AR04 | 0.67 |
| AR | Acceptance | AR05 | 0.67 |
| AR | Acceptance | AR06 | 0.76 |
| AR | Acceptance | AR07 | 0.62 |
| AR | Acceptance | AR08 | 0.71 |
| AR | Goal orientation | AR09 | 0.60 |
| AR | Goal orientation | AR10 | 0.73 |
| AR | Goal orientation | AR11 | 0.66 |
| AR | Goal orientation | AR12 | 0.78 |
| AR | Meaning orientation | AR13 | 0.74 |
| AR | Meaning orientation | AR14 | 0.75 |
| AR | Meaning orientation | AR15 | 0.80 |
| AR | Present-moment focus | AR16 | 0.81 |
| AR | Present-moment focus | AR17 | 0.77 |
| AR | Present-moment focus | AR18 | 0.65 |
| AR | Cognitive defusion | AR19 | 0.81 |
| AR | Cognitive defusion | AR20 | 0.56 |
| AR | Cognitive defusion | AR21 | 0.76 |
| AR | Cognitive defusion | AR22 | 0.54 |
| AR | Cognitive defusion | AR23 | 0.59 |
| AR | Cognitive defusion | AR24 | 0.66 |

*Note. Loadings are standardized estimates from the joint ten-factor confirmatory factor analysis using the WLSMV estimator. SEC = social and emotional competence; AR = academic resilience. Item codes correspond to the questionnaire. Values are rounded to two decimal places.*

**Supplementary Table S7. Spearman correlation matrix of the ten network dimensions**

| **Dimension** | **SRC** | **ORC** | **CRC** | **RDC** | **AW** | **AC** | **GO** | **MO** | **PMF** | **CD** |
| --- | --- | --- | --- | --- | --- | --- | --- | --- | --- | --- |
| SRC | - | 0.16 | 0.28 | 0.39 | 0.14 | 0.12 | 0.51 | 0.16 | 0.25 | 0.12 |
| ORC | 0.16 | - | 0.32 | 0.21 | 0.11 | 0.36 | 0.14 | 0.12 | 0.03 | 0.14 |
| CRC | 0.28 | 0.32 | - | 0.25 | 0.05 | 0.11 | 0.20 | 0.32 | 0.06 | 0.01 |
| RDC | 0.39 | 0.21 | 0.25 | - | 0.24 | 0.13 | 0.33 | 0.13 | 0.20 | 0.15 |
| AW | 0.14 | 0.11 | 0.05 | 0.24 | - | 0.20 | 0.23 | 0.13 | 0.19 | 0.21 |
| AC | 0.12 | 0.36 | 0.11 | 0.13 | 0.20 | - | 0.19 | 0.16 | 0.13 | 0.33 |
| GO | 0.51 | 0.14 | 0.20 | 0.33 | 0.23 | 0.19 | - | 0.24 | 0.33 | 0.19 |
| MO | 0.16 | 0.12 | 0.32 | 0.13 | 0.13 | 0.16 | 0.24 | - | 0.11 | 0.14 |
| PMF | 0.25 | 0.03 | 0.06 | 0.20 | 0.19 | 0.13 | 0.33 | 0.11 | - | 0.31 |
| CD | 0.12 | 0.14 | 0.01 | 0.15 | 0.21 | 0.33 | 0.19 | 0.14 | 0.31 | - |

*Note. Values are bivariate Spearman correlation coefficients among the ten dimension scores and are provided for descriptive purposes. They are not partial correlations; the regularized partial-correlation matrix is shown in Supplementary Figure S1. Node abbreviations are defined in Supplementary Table S1.*

**Supplementary Table S8. Definitions and representative items of the six academic resilience dimensions**

| **Dimension** | **Conceptual definition** | **Representative item (English translation)** | **Theoretical basis** |
| --- | --- | --- | --- |
| Awareness | Recognition and appraisal of academic demands, resources, and one's learning-related position. | “I am clear about the necessity of receiving a university education.” | Academic resilience process model; academic self-awareness |
| Acceptance | Recovering from academic setbacks, examining their causes, and acknowledging limitations in learning ability. | “After an academic setback, I recover quickly.” | Acceptance and Commitment Therapy; resilience process model |
| Goal orientation | Setting academic goals, planning, and sustaining effort toward those goals. | “I always have clear learning goals.” | Acceptance and Commitment Therapy; resilience process model |
| Meaning orientation | Finding value and meaning in learning processes, experiences, and demanding academic activities. | “Compared with the result, the learning process and the experience gained are also important to me.” | Acceptance and Commitment Therapy; resilience process model |
| Present-moment focus | Maintaining attention and involvement in the learning task currently being undertaken. | “I find it difficult to concentrate on the learning task I am currently doing.” | Present-moment awareness in Acceptance and Commitment Therapy |
| Cognitive defusion | Reduced interference from repetitive and unhelpful thoughts about academic difficulties and failure. | “Overthinking prevents me from making progress in my studies.” | Cognitive defusion in Acceptance and Commitment Therapy |

*Note. The definitions follow the operationalization used in Kong's University Student Academic Resilience Questionnaire. The questionnaire was administered in Chinese, and the representative items are author-provided English translations.* *Items 16–24 were reverse-scored before score calculation so that higher scores indicated greater present-moment focus and cognitive defusion.*

**Supplementary Table S9. Confirmatory factor analysis fit indices**

| **Model** | **χ²** | **df** | **p** | **χ²/df** | **CFI** | **TLI** | **RMSEA [90% CI]** | **SRMR** |
| --- | --- | --- | --- | --- | --- | --- | --- | --- |
| Four-factor social and emotional competence | 607.74 | 293 | <0.001 | 2.07 | 0.96 | 0.95 | 0.04 [0.03, 0.04] | 0.03 |
| Six-factor academic resilience | 748.36 | 237 | <0.001 | 3.16 | 0.93 | 0.92 | 0.05 [0.04, 0.05] | 0.04 |
| Joint ten-factor model | 1927.42 | 1130 | <0.001 | 1.71 | 0.94 | 0.93 | 0.03 [0.03, 0.03] | 0.03 |
| Nine-factor model merging self-related competence and goal orientation | 2397.97 | 1139 | <0.001 | 2.11 | 0.91 | 0.90 | 0.04 [0.04, 0.04] | 0.04 |

*Note. chi-square = model chi-square; df = degrees of freedom; CFI = comparative fit index; TLI = Tucker-Lewis index; RMSEA = root mean square error of approximation; CI = confidence interval; SRMR = standardized root mean square residual. All model chi-square tests had p < 0.001.*

**Supplementary Table S10. Composite reliability, average variance extracted, and standardized loading ranges**

| **Dimension** | **CR** | **AVE** | **Standardized loading range** |
| --- | --- | --- | --- |
| Self-related competence | 0.87 | 0.53 | 0.60-0.86 |
| Other-related competence | 0.82 | 0.48 | 0.59-0.81 |
| Collective-related competence | 0.87 | 0.47 | 0.60-0.75 |
| Responsible decision-making competence | 0.87 | 0.48 | 0.65-0.82 |
| Awareness | 0.77 | 0.46 | 0.59-0.79 |
| Acceptance | 0.79 | 0.48 | 0.62-0.76 |
| Goal orientation | 0.79 | 0.48 | 0.60-0.78 |
| Meaning orientation | 0.81 | 0.58 | 0.74-0.80 |
| Present-moment focus | 0.79 | 0.55 | 0.65-0.81 |
| Cognitive defusion | 0.82 | 0.44 | 0.54-0.81 |

*Note. CR = composite reliability; AVE = average variance extracted. Seven dimensions had AVE values below 0.50: other-related competence, collective-related competence, responsible decision-making competence, awareness, acceptance, goal orientation, and cognitive defusion.*

**Supplementary Table S11. Sensitivity analyses**

| **Model** | **Nonzero edges** | **Largest cross-construct edge** | **Node with highest strength** | **Highest bridge-strength SEC node** | **Edge-weight correlation** |
| --- | --- | --- | --- | --- | --- |
| Main model | 28 | Self-related competence-goal orientation | Goal orientation | Self-related competence | - |
| Spearman, gamma = 0.25 | 29 | Self-related competence-goal orientation | Goal orientation | Self-related competence | 0.995 |
| Spearman, gamma = 0.75 | 28 | Self-related competence-goal orientation | Goal orientation | Self-related competence | 1.000 |
| Spearman, gamma = 1.00 | 28 | Self-related competence-goal orientation | Goal orientation | Self-related competence | 1.000 |
| Pearson, gamma = 0.50 | 26 | Self-related competence-goal orientation | Goal orientation | Self-related competence | 0.985 |
| Covariate- and university-source-adjusted | 29 | Self-related competence-goal orientation | Goal orientation | Self-related competence | 0.993 |
| Self-related competence items 5 and 6 removed | 29 | Self-related competence-goal orientation | Goal orientation | Self-related competence | 0.994 |
| Unregularized Spearman partial-correlation network | 45 | Self-related competence-goal orientation | Goal orientation | Self-related competence | 0.968 |

*Note. Edge-weight correlation is Spearman's rank correlation between the 45 corresponding estimates in each sensitivity model and the primary network. Alternative gamma rows use different EBIC tuning values. The unregularized model contains partial correlations calculated from the inverse Spearman correlation matrix without penalization or edge selection. The item-deletion model removes items 5 and 6 from self-related competence. Detailed leave-one-university-out results are reported in Supplementary Table S3.* *Detailed cross-construct edge weights and ranks are reported in Supplementary Table S15. Nonzero estimates in the unregularized network do not by themselves indicate statistical significance.*

**Supplementary Table S12. Node strength and bridge strength in the primary EBICglasso network**

| **Module** | **Dimension** | **Strength** | **Bridge strength** |
| --- | --- | --- | --- |
| Social and emotional competence | Self-related competence | 0.80 | 0.44 |
| Social and emotional competence | Other-related competence | 0.60 | 0.29 |
| Social and emotional competence | Collective-related competence | 0.73 | 0.26 |
| Social and emotional competence | Responsible decision-making competence | 0.70 | 0.31 |
| Academic resilience | Awareness | 0.48 | — |
| Academic resilience | Acceptance | 0.72 | — |
| Academic resilience | Goal orientation | 0.95 | — |
| Academic resilience | Meaning orientation | 0.49 | — |
| Academic resilience | Present-moment focus | 0.57 | — |
| Academic resilience | Cognitive defusion | 0.66 | — |

*Note. Strength and bridge strength are unstandardized estimates from the primary EBICglasso network. Bridge-strength comparisons in the main text focus on the social and emotional competence dimensions because the two modules contain different numbers of nodes.*

**Supplementary Table S13. Nonzero retention of cross-construct edges**

| SEC dimension | AR dimension | Nonzero retention |
| --- | --- | --- |
| Self-related competence | Goal orientation | 1.00 |
| Other-related competence | Acceptance | 1.00 |
| Collective-related competence | Meaning orientation | 1.00 |
| Responsible decision-making competence | Awareness | 1.00 |
| Responsible decision-making competence | Goal orientation | 1.00 |
| Self-related competence | Present-moment focus | 0.99 |
| Responsible decision-making competence | Present-moment focus | 0.91 |
| Collective-related competence | Cognitive defusion | 0.69 |
| Responsible decision-making competence | Cognitive defusion | 0.73 |

*Note. Nonzero retention is the proportion of 5,000 nonparametric bootstrap networks in which an edge was estimated as nonzero. Values are descriptive stability indicators.*

**Supplementary Table S14. Associations between academic resilience scores and self-reported academic rank**

| Academic resilience score | N | Spearman’s ρ | 95% bootstrap CI | Unadjusted p | BH-adjusted p |
| --- | --- | --- | --- | --- | --- |
| Total score | 1,139 | 0.294 | [0.238, 0.348] | <0.001 | — |
| Awareness | 1,139 | 0.165 | [0.108, 0.221] | <0.001 | <0.001 |
| Acceptance | 1,139 | 0.096 | [0.037, 0.153] | <0.001 | <0.001 |
| Goal orientation | 1,139 | 0.410 | [0.359, 0.458] | <0.001 | <0.001 |
| Meaning orientation | 1,139 | 0.108 | [0.050, 0.163] | <0.001 | <0.001 |
| Present-moment focus | 1,139 | 0.173 | [0.117, 0.230] | <0.001 | <0.001 |
| Cognitive defusion | 1,139 | 0.126 | [0.067, 0.181] | <0.001 | <0.001 |

*Note. Academic rank refers to participants’ self-reported position within their major during the previous academic year and was coded from 1 (bottom 25%) to 4 (top 25%). The 52 “not sure” responses were excluded from these analyses only. Confidence intervals were obtained from 5,000 paired-case percentile bootstrap samples at the individual level (random seed = 20260911). Tests were two-sided and used asymptotic p values. The total-score association was the primary analysis; Benjamini–Hochberg adjustment was applied to the six secondary dimension-level tests. Confidence intervals were not adjusted for multiple comparisons.*

**Supplementary Table S15. Cross-construct edge weights and absolute-weight ranks across network specifications**

| Edge | γ = 0.50 | γ = 0.25 | γ = 0.75 | γ = 1.00 | Unregularized |
| --- | --- | --- | --- | --- | --- |
| SRC–AW | 0 (—) | 0 (—) | 0 (—) | 0 (—) | −0.0236 (15) |
| ORC–AW | 0 (—) | 0 (—) | 0 (—) | 0 (—) | 0.0167 (19) |
| CRC–AW | 0 (—) | 0 (—) | 0 (—) | 0 (—) | −0.0460 (10) |
| RDC–AW | 0.1325 (4) | 0.1347 (4) | 0.1325 (4) | 0.1325 (4) | 0.1601 (4) |
| SRC–AC | 0 (—) | 0 (—) | 0 (—) | 0 (—) | −0.0027 (24) |
| ORC–AC | 0.2861 (2) | 0.2909 (2) | 0.2861 (2) | 0.2861 (2) | 0.3165 (2) |
| CRC–AC | 0 (—) | 0 (—) | 0 (—) | 0 (—) | −0.0257 (14) |
| RDC–AC | 0 (—) | 0 (—) | 0 (—) | 0 (—) | −0.0233 (16) |
| SRC–GO | 0.3704 (1) | 0.3731 (1) | 0.3704 (1) | 0.3704 (1) | 0.3878 (1) |
| ORC–GO | 0 (—) | 0 (—) | 0 (—) | 0 (—) | −0.0041 (23) |
| CRC–GO | 0 (—) | 0 (—) | 0 (—) | 0 (—) | 0.0137 (20) |
| RDC–GO | 0.1144 (5) | 0.1144 (5) | 0.1144 (5) | 0.1144 (5) | 0.1118 (5) |
| SRC–MO | 0 (—) | 0 (—) | 0 (—) | 0 (—) | −0.0108 (21) |
| ORC–MO | 0 (—) | 0 (—) | 0 (—) | 0 (—) | −0.0315 (13) |
| CRC–MO | 0.2383 (3) | 0.2427 (3) | 0.2383 (3) | 0.2383 (3) | 0.2803 (3) |
| RDC–MO | 0 (—) | 0 (—) | 0 (—) | 0 (—) | −0.0190 (17) |
| SRC–PMF | 0.0707 (6) | 0.0725 (6) | 0.0707 (6) | 0.0707 (6) | 0.0903 (6) |
| ORC–PMF | 0 (—) | −0.0079 (10) | 0 (—) | 0 (—) | −0.0597 (8) |
| CRC–PMF | 0 (—) | 0 (—) | 0 (—) | 0 (—) | −0.0169 (18) |
| RDC–PMF | 0.0398 (7) | 0.0417 (7) | 0.0398 (7) | 0.0398 (7) | 0.0538 (9) |
| SRC–CD | 0 (—) | 0 (—) | 0 (—) | 0 (—) | −0.0076 (22) |
| ORC–CD | 0 (—) | 0 (—) | 0 (—) | 0 (—) | 0.0325 (12) |
| CRC–CD | −0.0249 (8) | −0.0358 (8) | −0.0249 (8) | −0.0249 (8) | −0.0815 (7) |
| RDC–CD | 0.0207 (9) | 0.0235 (9) | 0.0207 (9) | 0.0207 (9) | 0.0425 (11) |

*Note. Entries are signed edge weights, with absolute-weight ranks among all 24 cross-construct edges in parentheses. All models used the same 1,191 participants, ten dimension scores, and Spearman correlation matrix. The primary model used γ = 0.50. Ranks and nonzero status were determined from unrounded estimates; average ranks were used for ties, and ranks are omitted for zero edges. A zero indicates that the regularized model did not retain the edge. Unregularized estimates were obtained from the inverse correlation matrix without penalization or edge selection and are not presented as significance tests. SRC = self-related competence; ORC = other-related competence; CRC = collective-related competence; RDC = responsible decision-making competence; AW = awareness; AC = acceptance; GO = goal orientation; MO = meaning orientation; PMF = present-moment focus; CD = cognitive defusion.*

**Supplementary Figure S1. Partial-correlation matrix of the ten network dimensions.**

*Note. Values are partial correlation coefficients estimated using EBICglasso. Blue indicates positive partial associations and red indicates negative partial associations. Color intensity represents the absolute edge weight. The diagonal is omitted. SRC = self-related competence; ORC = other-related competence; CRC = collective-related competence; RDC = responsible decision-making competence; AW = awareness; AC = acceptance; GO = goal orientation; MO = meaning orientation; PMF = present-moment focus; CD = cognitive defusion.*

**Supplementary Figure S2. Bootstrapped difference test among the nine retained cross-construct edge weights.**

*Note.* Diagonal values are edge-weight point estimates. Blue cells indicate pairwise bootstrap difference intervals that did not span zero; grey cells indicate intervals that spanned zero. These exploratory comparisons were interpreted cautiously because multiple pairwise tests were conducted. SRC = self-related competence; ORC = other-related competence; CRC = collective-related competence; RDC = responsible decision-making competence; AW = awareness; AC = acceptance; GO = goal orientation; MO = meaning orientation; PMF = present-moment focus; CD = cognitive defusion.

**Supplementary Figure S3. Bootstrapped strength-centrality difference test.**

*Note. Diagonal values are unstandardized strength point estimates. Blue cells indicate pairwise bootstrap difference intervals that did not span zero; grey cells indicate intervals that spanned zero. These exploratory comparisons were interpreted cautiously because multiple pairwise tests were conducted. Node abbreviations are defined in Supplementary Figure S2.*

**Supplementary Figure S4. Bootstrapped bridge-strength difference test within the social and emotional competence module.**

*Note.* Diagonal values are unstandardized bridge-strength point estimates. Blue cells indicate pairwise bootstrap difference intervals that did not span zero; grey cells indicate intervals that spanned zero. Comparisons were limited to the four social and emotional competence nodes and were interpreted as exploratory. SRC = self-related competence; RDC = responsible decision-making competence; ORC = other-related competence; CRC = collective-related competence.

**Supplementary Figure S5. Case-dropping bootstrap stability of (A) strength centrality and (B) bridge-strength centrality.**

*Note. Panel A shows strength centrality and Panel B bridge-strength centrality. The x-axis shows the proportion of cases retained, and the y-axis shows the mean correlation with the original-sample estimate. For both indices, CS(cor = 0.70) = 0.75, the maximum case-dropping proportion tested.*
